# Influence of a hyperglycaemic diet on *Trypanosoma cruzi* infection in mice model

**DOI:** 10.1590/0074-02760250092

**Published:** 2025-12-15

**Authors:** Aline Coelho das Mercês, Flávia de Souza Marques, Bruno Teixeira Martins, Gabriel José Lucas Moreira, Bruno Mendes Roatt, Cláudia Martins Carneiro, Silvia de Paula-Gomes, Joana Ferreira do Amaral, Paula Melo de Abreu Vieira

**Affiliations:** 1 Universidade Federal de Ouro Preto, Instituto de Ciências Exatas e Biológicas, Núcleo de Pesquisas em Ciências Biológicas, Departamento de Ciências Biológicas, Laboratório de Morfopatologia, Ouro Preto, MG, Brasil; 2 Universidade Federal de Ouro Preto, Instituto de Ciências Exatas e Biológicas, Núcleo de Pesquisas em Ciências Biológicas, Laboratório de Imunopatologia, Ouro Preto, MG, Brasil; 3 Universidade Federal de Ouro Preto, Escola de Farmácia, Departamento de Análises Clínicas, Ouro Preto, MG, Brasil; 4 Universidade Federal de Ouro Preto, Instituto de Ciências Exatas e Biológicas, Núcleo de Pesquisas em Ciências Biológicas, Laboratório de Bioquímica e Biologia Molecular, Ouro Preto, MG, Brasil; 5 Universidade Federal de Ouro Preto, Laboratório Multiusuário de Bioquímica Nutricional, Ouro Preto, MG, Brasil

**Keywords:** Chagas disease, Trypanosoma cruzi, metabolic diseases

## Abstract

**BACKGROUND:**

Parasitic diseases may increase the risk of metabolic abnormalities through persistent inflammation. However, the effects of a hyperglycaemic diet during *Trypanosoma cruzi* infection remain poorly understood.

**OBJECTIVES:**

This study aimed to investigate the metabolic, parasitological, immunological, and histological effects of a hyperglycaemic diet during acute *T. cruzi* infection in mice.

**METHODS:**

C57BL/6 mice were divided into four groups: non-infected with standard diet (NISD), infected with a standard diet (ISD), non-infected with hyperglycaemic diet (NIHD), and infected with hyperglycaemic diet (IHD). Animals were fed their respective diets for eight weeks prior to infection and monitored up to 30 days post-infection (DPI) for blood glucose, body mass, biochemical markers, parasitaemia, tissue alterations, and immune cell profiles.

**FINDINGS:**

At the time of infection, hyperglycaemic diet groups showed higher blood glucose and body mass. By 30 DPI, these animals exhibited lower glucose, increased parasitaemia, adipose tissue hypertrophy, and reduced cholesterol levels compared with controls. Infected groups showed an increased CD4+ IFN-γ+ T cells at 10 DPI, whereas macrophage expansion was observed only in ISD mice. Cardiac parasitism was higher at 30 DPI than at 10 DPI.

**MAIN CONCLUSIONS:**

These results show that *T. cruzi* infection affects metabolic parameters and that a hyperglycaemic diet worsens parasitological outcomes during the acute phase of infection and appears to downregulate the immune response.

Discovered by Carlos Chagas in 1909, Chagas disease (CD), or American trypanosomiasis, is caused by the protozoan *Trypanosoma cruzi*, transmitted primarily by the haematophagous insect vector known as the triatomine or “kissing bug”.^1^ In addition to vector transmission, other routes of infection include congenital transmission, blood transfusion, organ transplantation, oral transmission, and laboratory accidents.^2^ Epidemiologically, CD is considered one of the major neglected tropical diseases, endemic in 21 Latin American countries, with a significant global impact. It is estimated that over 7 million people are affected by the disease, with 100 million at risk of contracting it. However, less than 10% of cases are diagnosed, contributing to an alarming number of disease-related deaths.^3^

Demographic changes and lifestyle habits are contributing to the rise of non-communicable chronic diseases, such as obesity and diabetes mellitus, which have become among the leading causes of morbidity and mortality globally, highlighting the need for a comprehensive approach to CD control.^4^ ^,^ ^5^ ^,^ ^6^ A hyperglycaemic diet is characterised by the consumption of foods with a high glycaemic index, resulting in high glycaemic loads. This leads to elevated insulin levels, which activate fat synthesis and increase triacylglycerol concentrations in the blood. Such a diet can cause metabolic disturbances, including increased body mass and elevated serum levels of insulin and triacylglycerol.^7^ The metabolic changes induced by a hyperglycaemic diet also affect the immune response, particularly in conditions such as obesity. The enlargement of adipocytes can lead to the release of pro-inflammatory substances, promoting the infiltration of immune cells, such as macrophages, into affected areas of adipose tissue. This inflammatory state may contribute to the progression of health conditions associated with obesity.^8^ ^,^ ^9^

The relationship between carbohydrate consumption and parasitic diseases, including CD, is an area that remains underexplored in the scientific literature. However, studies have demonstrated a link between a hyperglycaemic diet and conditions such as obesity and type 2 diabetes mellitus, which are often observed in CD patients. For example, research assessing the prevalence of metabolic syndrome in CD patients revealed high rates of obesity, diabetes mellitus, and dyslipidaemia in this population.^10^

The accumulation of body adipose tissue and elevated blood cholesterol levels are particularly relevant for CD patients, as adipose tissue can serve as a reservoir for *T. cruzi*. This reservoir contributes to an increased parasite load and can lead to insulin resistance, thereby triggering a chronic inflammatory state. Additionally, the imbalance in the regulation of pro-inflammatory and anti-inflammatory cytokines may increase the risk of tissue damage in the host.^11^ ^,^ ^12^

A study investigating the influence of a hyperglycaemic diet on *T. cruzi* infection could clarify how diet-induced metabolic changes affect the immune response and the progression of the infection. This would help to better understand the underlying mechanisms of the interaction between diet and parasitic infection, offering insights into potential therapeutic and preventive strategies. In this context, the aim of the present work was to investigate the influence of a hyperglycaemic diet on *T. cruzi* infection in a murine model.

## MATERIALS AND METHODS

*Animals and experimental groups* - Thirty-six isogenic male C57BL/6 mice, 30 days old, born at the Animal Science Centre of the Federal University of Ouro Preto (CCA - UFOP), were used.

The animals were randomly assigned to four experimental groups: non-infected animals subjected to a standard diet (NISD, n = 8), *T. cruzi*-infected animals subjected to a standard diet (ISD, n = 10), non-infected animals subjected to a hyperglycaemic diet (NIHD, n = 8), and *T. cruzi*-infected animals subjected to a hyperglycaemic diet (IHD, n = 10). Each group was further subdivided equally for evaluation at 10 and 30 days post-infection (DPI).

All experimental procedures were conducted in accordance with the ethical principles recommended by the National Council for the Control of Animal Experimentation (CONCEA) and were approved by the Animal Use Ethics Committee of the Federal University of Ouro Preto (CEUA - UFOP) under protocol no. 8981260122.

*Diet composition* - The standard diet was formulated according to the AIN-93G diet^13^ with the aim of maintaining the animals’ nutritional status. The hyperglycaemic diet was prepared according to the HFrD diet,^14^ a modified version of the AIN-93 diet, designed to induce metabolic changes related to hyperglycaemic eating habits. The composition of the diets is detailed in Table I.

**TABLE I:** Composition of standard and hyperglycemic diets

Ingredients/Kg	Standard diet (g)	Hyperglycemic diet (g)
Corn starch	397,49	
Fructose	-	553
Casein	200	140
Maltodextrin	132	-
Sucrose	100	12
Soybean oil	70	40
Cellulose	50	50
Mineral mix^*a*^	35	35
Vitamin mix^*b*^	10	10
L-cystine	3	1,8
Choline bitartrate	2,5	2,5
Lard	-	154,1
Tert-butylhydroquinone	14	-
BHT preservative	2	2

*a*: mineral mix (g/kg): NaCl - 139.3; KI - 0.79; MgSO4.7H2O - 57.3; CaCO3 - 381.4; MnSO4.H2O - 4.01; FeSO4.7H2O - 0.548; CuSO4.5H2O - 0.477; CoCl2.6H2O - 0.023; KH2PO4 - 389.0; *b*: vitamin mix (mg/kg): retinol acetate - 690; cholecalciferol - 5; P-aminobenzoic acid - 10,000; inositol - 10,000; niacin - 4,000; riboflavin - 800; thiamine HCl - 500; folic acid - 200; biotin - 40; cyanocobalamin - 3; dl-α-tocopherol - 6,700; sucrose - q.s.p. 1,000.

*Experimental strategy* - Throughout the experiment, the animals were provided with water *ad libitum*. Eight weeks prior to the day of infection, mice were subjected to their respective diets. The infection was carried out 56 days after the introduction of the diet (0 DAI). Euthanasia was performed at 10 days after infection (10 DAI) to assess a period close to the peak of parasitaemia, while euthanasia was conducted at 30 DAI to evaluate the period following parasitaemia.

*Trypanosoma cruzi infection* - The animals were inoculated intraperitoneally with 1x10⁴ bloodstream trypomastigotes forms of Colombian strain of *T. cruzi*. These forms were obtained from mice previously infected with the same strain, also via intraperitoneal injection, for strain maintenance by the Morphopathology Laboratory at the UFOP, with passages performed every 15 days in *Swiss* mice.

*Survive rate* - To determine survival rate, animals were monitored daily until the day of euthanasia. Deaths were recorded and expressed as a cumulative percentage.

*Parasitaemia curve* - After the 4th day of infection mice were evaluated daily to establish the parasitaemia curve according to Brener’s adapted methodology.^15^ In short, five microliters of blood were taken from the caudal vein and placed on a slide. Blood trypomastigotes were counted in 50 random microscopic fields in an optical microscope. This procedure was repeated daily until no parasites were observed for five consecutive days. Results were given as parasites/0.1 mL of blood.

*Ingestion quantification* - The animals’ food intake was measured weekly. At the start of the diet, 105 g of food was provided. After each week, the remaining food was weighed, and the amount was replenished to 105 g using an SF-400 digital scale.

*Body mass quantification* - The animals were weighed before the start of the diet and on the day of euthanasia (10 and 30 DPI) using an SF-400 digital scale.

*Euthanasia and samples* - Four animals from the non-infected groups and five from the infected groups were euthanised at 10 and 30 DPI. Euthanasia was performed under general anaesthesia, with the administration of ketamine (90 mg/kg) and xylazine hydrochloride (9 mg/kg), followed by exsanguination, which was carried out after confirming the absence of reflexes and resistance to painful stimuli in the mice.

At euthanasia, 200 µL of blood was collected from the brachial plexus and centrifuged at 10,000 rpm for 10 min at 4ºC. The serum was then collected for biochemical analyses.

*Tissue collection* - At necropsy, fragments of the heart and adipose tissue were collected for histological analysis and quantification of tissue parasitism. All retroperitoneal and epididymal adipose tissue was removed and weighed. The spleen was collected for cellular phenotypic characterisation. The tibia was collected, cleaned, and measured using a caliper to normalise adipose tissue weight to tibia length.

*Biochemical analyses* - Serum insulin levels were quantified using the Rat/Mouse Insulin ELISA EZRMI-13K kit (Sigma-Aldrich), following the manufacturer’s instructions. Cholesterol was measured using the Monoreagent Cholesterol kit (Bioclin, K083), following the manufacturer’s recommendations, with the following adaptation for the animal model: 300 μL of Reagent No. 1. Triglycerides were measured using the Monoreagent Triglycerides kit (Bioclin, K117), with the following adaptation: 300 μL of Reagent No. 1. Capillary blood glucose was measured from the tail vein of mice fasted for 12 hours using a glucometer (On Call Plus II) at two time points: prior to the introduction of the diet and on the day of euthanasia.

*Spleen immunophenotyping* - After spleen collection, the samples were mechanically dissociated in incomplete RPMI medium using a scalpel to obtain a single-cell suspension. Subsequent steps - including cell concentration adjustment, viability staining, surface antibody staining, red blood cell lysis, intracellular cytokine staining (Table II), fixation, and storage - were performed as previously described by Vieira.^16^

**TABLE II:** Panel of monoclonal antibodies used in cell immunophenotyping assays and intracellular cytokine staining in the *ex vivo* context

Marke^r^*	Fluorochrome	Clone	Dilution	Function
CD3e	BV650	17A2	1:100	Defines T lymphocytes
CD4	BV605	RM4-5	1:200	Defines subpopulation of helper T lymphocytes
CD8a	BV786	53-6.7	1:100	Defines subpopulation of cytotoxic T lymphocytes
CD11b	FITC	M1-70	1:80	Defines macrophage subpopulation
TNF	PE-Cy7	LG.3A10	1:100	Beltcin type 1
IFN-γ	PE	XMGI1.2	1:50	Beltcin type 1
IL-10	BV711	JES5-16E3	1:50	Immunomodulatory beltcin

*all markers used were from the BD bioscience brand.

**Fig. 1:**
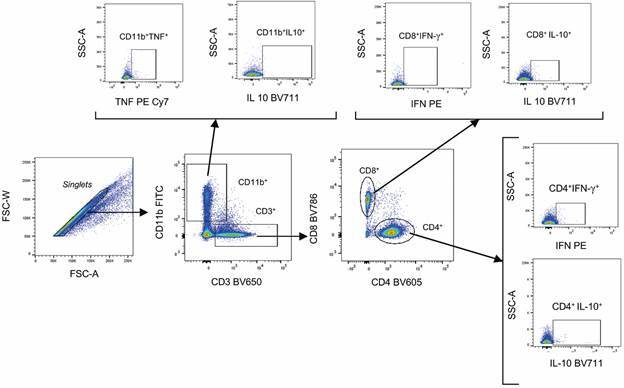
proposed analysis strategy for the phenotypic characterisation of the percentage of total lymphocyte populations and their subpopulations (CD4⁺, CD8⁺, and CD11b⁺) producing the cytokines IFN-γ, TNF, and IL-10 from *ex vivo* spleen samples.

A minimum of 100,000 events per sample were acquired using an LSR Fortessa flow cytometer (BD Biosciences), operated using BD FACSDiva™ software. Instrument compensation was performed using BD™ CompBeads. Dead or apoptotic cells were excluded based on FVS450 staining, which binds to surface and intracellular amines. Data were analysed using FlowJo™ v10.8.0 software (BD Biosciences). The gating strategy for characterising total lymphocytes and their subpopulations (CD4⁺, CD8⁺, and CD11b⁺) producing interferon (IFN)-γ, tumour necrosis factor (TNF), and interleukin (IL)-10 is shown in Fig. 1.

*Parasite load quantification* - Heart and adipose tissue fragments, previously stored at -80ºC, were dissected with a scalpel and weighed to approximately 30 mg. Samples were transferred to 1.5 mL microtubes, and DNA extraction was performed using the Wizard^®^ Genomic DNA Purification Kit (Promega, Madison, WI, USA), following the manufacturer’s instructions with minor modifications, based on the protocol described by Marques et al.^17^ DNA concentration and purity were assessed using a NanoDrop 2000 spectrophotometer (Thermo Scientific, USA), and samples were stored at -20ºC until quantitative polymerase chain reaction (qPCR) analysis.

*Histopathological evaluation* - At necropsy, portions of the heart and adipose tissue were fixed in methanol/dimethylsulfoxide (80/20) and stored at 4ºC, with the fixation solution replaced daily for three consecutive days. The tissue fragments were then routinely dehydrated through an ascending series of alcohols and cleared in xylene. Subsequently, the tissues were embedded in paraffin blocks and sectioned at 4 µm thickness for slide preparation. Afterward, slides were stained with haematoxylin and eosin (H&E), dried in an oven at approximately 50ºC, and once completely dried, coverslips were mounted using Entellan^®^.

*Morphometric analysis* - Inflammation was quantified in the heart, a target organ for the strain employed, and in adipose tissue, which has been described as a parasitic reservoir and is directly influenced by insulin. A semi-automated digital microscope (Leica DM5000B) equipped with a 5 MegaPixel CCD camera, model MC170HD, was used to capture images of the slides at 40× magnification. For the heart, 25 random fields were imaged, while for adipose tissue, 15 random fields were captured.

Following image acquisition, the cellular nuclei were quantified with Leica Qwin v3.5.1 software.

Both the microscope and the analysis software are part of the Advanced Microscopy and Microanalysis Multi-User Laboratory (LMU-MAM) at the Biological Sciences Research Centre (NUPEB), UFOP.

*Statistical analysis* - Statistical tests were performed using GraphPad Prism 8.0.2 software (Prism Software, Irvine, CA, USA). The Shapiro-Wilk test was conducted to confirm normality. For parametric data, comparisons between groups were performed using Student’s t-test or one-way or two-way analysis of variance (ANOVA). When significant differences were detected, Tukey’s or Bonferroni’s test was applied to determine specific differences between means. For non-parametric data, the Mann-Whitney test was conducted. Differences between means were considered significant at p < 0.05.

## RESULTS

**Fig. 2:**
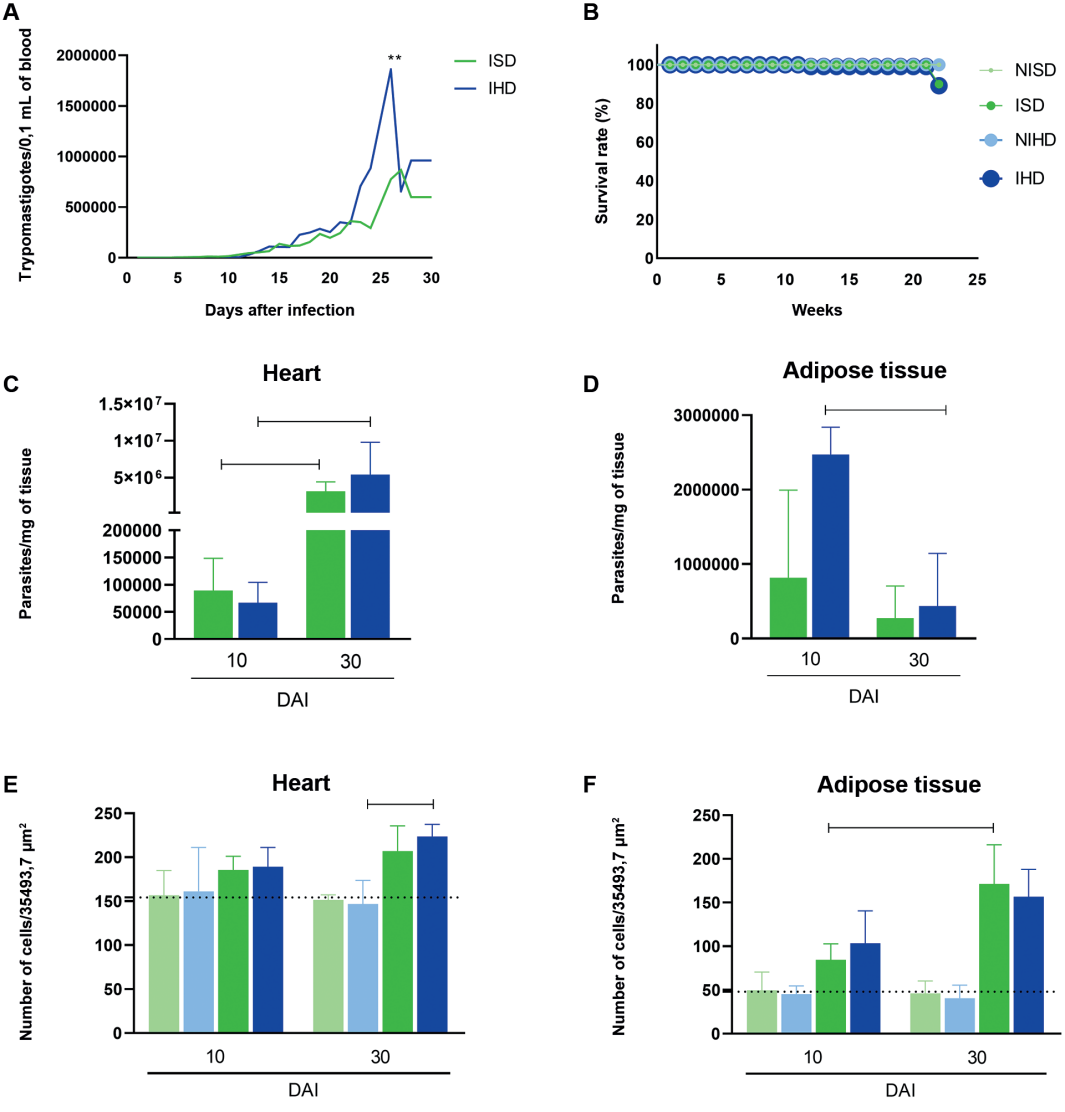
impact of infection on C57BL/6 mice, either non-infected or infected with the Colombian strain of *Trypanosoma cruzi*, and subjected to a standard diet (SD) or a hyperglycaemic diet (HD) in the following groups: non-infected animals subjected to a standard diet (NISD), *T. cruzi*-infected animals subjected to a standard diet (ISD), non-infected animals subjected to a hyperglycaemic diet (NIHD), and *T. cruzi*-infected animals subjected to a hyperglycaemic diet (IHD). (A) Parasitaemia. Each curve represents the mean of 20 animals from each infected group over 30 days after infection (DAI). (B) Survival rate. Each curve represents the mean of 36 animals from each non-infected or infected group over 30 days post-infection. (C) Number of parasites in the heart. The “bar” indicates a difference between infected groups at 30 DAI compared with the same groups at 10 DAI. (D) Number of parasites in adipose tissue. The “bar” represents a decrease in tissue parasitism in the IHD group at 30 DAI compared with 10 DAI. (E) Quantification of the inflammatory process in the heart. The “bar” denotes an increase in inflammation in the infected group subjected to a hyperglycaemic diet at 30 DAI compared with its respective control. (F) Quantification of the inflammatory process in adipose tissue. The “bar” represents an increase in inflammation in the group subjected to a standard diet at 30 DAI compared with the same group at 10 DAI. Values are expressed as mean ± standard deviation.

*Impact of diet on disease progression and tissue pathology in the experimental model* - The higher parasitaemia observed in the IHD group on days 25 and 26 (Fig. 2A) may be associated with the reduced survival in this group (Fig. 2B). While all non-infected animals survived, infected animals on the hyperglycaemic diet (IHD) exhibited lower survival (80%) compared with those on the standard diet (90%), suggesting that the increased parasite burden may contribute to disease severity and mortality.

The impact of disease progression was also evaluated by quantifying parasitism in the heart and adipose tissue. In the heart, consumption of the hyperglycaemic diet appeared to result in a higher parasite burden at 10 days after infection, which was the opposite profile to that observed in the IHD group, where a significant increase was only detected at 30 days after infection (Fig. 2C). Morphometric analysis of cardiac inflammation further revealed that, at 30 DAI, the infected group on a hyperglycaemic diet (IHD 30 DAI) exhibited a more pronounced inflammatory response compared with the non-infected group on the same diet (NIHD 30 DAI) (Fig. 2E). This indicates that the inflammatory process in the heart is associated with disease progression and the earlier increase in parasite burden.

In adipose tissue, group differences underscored a dietary influence. Higher parasite levels were observed in the IHD group at 10 DAI compared with the IHD group at 30 DAI (Fig. 2D). However, unlike the findings in the heart, the higher parasite burden at day 10 did not influence the inflammatory response, as only the ISD group displayed a significant increase at 30 DAI (Fig. 2F). These findings suggest that hyperglycaemia may modulate parasite persistence and distribution, with subsequent effects on tissue-specific inflammation in the heart, which correlates with the observed parasitaemia and survival rates.

**Fig. 3:**
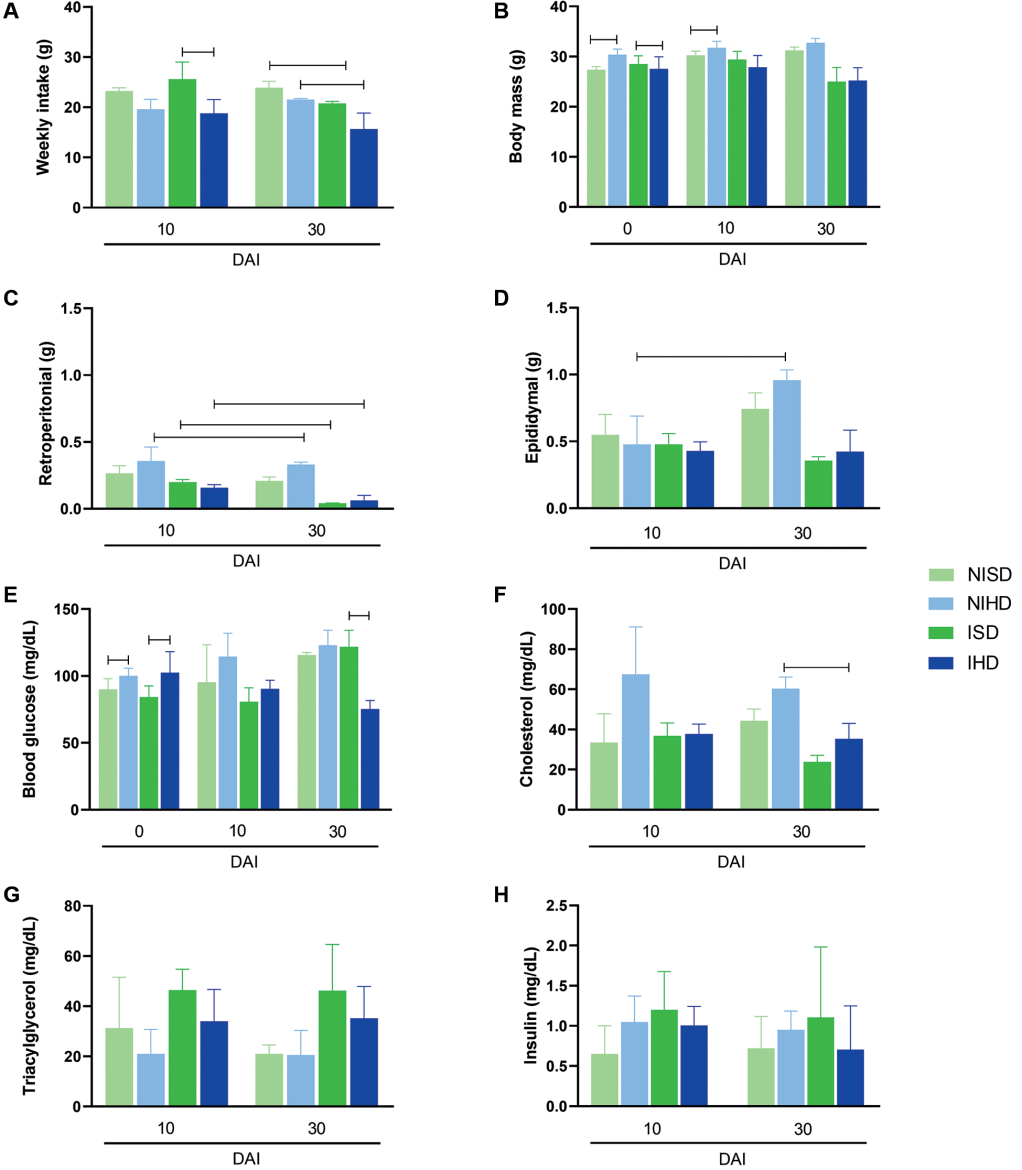
impact of infection and/or diet on food consumption, body weight, and metabolic profile in C57BL/6 mice, either non-infected or infected with the Colombian strain of *Trypanosoma cruzi*, and subjected to a standard diet (SD) or a hyperglycaemic diet (HD) in the following groups: non-infected animals subjected to a standard diet (NISD), *T. cruzi*-infected animals subjected to a standard diet (ISD), non-infected animals subjected to a hyperglycaemic diet (NIHD), and *T. cruzi*-infected animals subjected to a hyperglycaemic diet (IHD). (A) Food intake quantification. The “bar” indicates statistically significant differences between infected groups at 10 days after infection (DAI) and between infected groups and their respective controls at 30 DAI (p < 0.05). (B) Body mass. The “bar” denotes statistically significant differences between non-infected and infected groups prior to infection and between non-infected groups at 10 DAI. (C) Retroperitoneal adipose tissue mass. The “bar” indicates a decrease in retroperitoneal adipose tissue content in the NIHD group at 30 DAI compared to 10 DAI, as well as a decrease in both infected groups at 30 DAI compared to 10 DAI. (D) Epididymal adipose tissue mass. The “bar” represents an increase in epididymal adipose tissue content in the NIHD group at 30 DAI compared to 10 DAI. (E) Blood glucose quantification. The “bar” denotes higher glucose levels in mice subjected to a hyperglycaemic diet compared to those on a standard diet prior to infection, and a decrease in glucose levels in the IHD group compared to the ISD group at 30 DAI. (F) Serum cholesterol levels. The “bar” represents a reduction in cholesterol levels in infected mice subjected to a hyperglycaemic diet compared to their respective controls, as well as a difference between the NIHD and IHD groups at 30 DAI. (G) Triacylglycerol levels. (H) Serum insulin levels. Values are expressed as mean ± standard deviation.

*Impact of diet and T. cruzi infection on food intake, body composition, and metabolic parameters in mice* - To assess the effects of diet and infection, mice were monitored weekly for food intake, body mass, retroperitoneal and epididymal adipose tissue mass, blood glucose, serum insulin, triacylglycerol, and total cholesterol (Fig. 3).

The analysis of weekly food intake revealed that infected animals, irrespective of diet, exhibited a significant reduction in consumption at 30 DAI compared with their respective controls. This indicates that long-term infection itself influenced feeding behaviour. Furthermore, at both 10 and 30 DAI, diet also affected intake, with the IHD group consuming less food than the ISD group (Fig. 3A). These findings are consistent with body mass measurements, as non-infected mice on the hyperglycaemic diet displayed higher body mass both at the time of infection and at 10 DAI (Fig. 3B).

The analysis of retroperitoneal adipose tissue further highlighted the impact of infection, potentially linked to food intake, as infected groups showed higher retroperitoneal adipose tissue at 10 DAI relative to 30 DAI (Fig. 3C). Diet also significantly influenced this parameter, with non-infected mice on the hyperglycaemic diet displaying higher retroperitoneal adipose tissue at both 10 and 30 DAI. Similarly, diet appeared to affect epididymal adipose tissue accumulation, as non-infected mice on the hyperglycaemic diet showed increased epididymal adipose tissue at 30 DAI compared with 10 DAI (Fig. 3D).

The effects of food intake and long-term infection were also evident in biochemical analyses. Before infection, mice fed a hyperglycaemic diet exhibited higher blood glucose levels than those on a standard diet. By 30 DAI, however, this trend had reversed, with infected animals on the standard diet showing altered glucose levels (Fig. 3E). At 30 DAI, cholesterol levels were higher in non-infected mice on a hyperglycaemic diet than in infected animals, indicating that infection distinctly affected cholesterol metabolism (Fig. 3F). No significant differences were observed in triacylglycerol levels (Fig. 3G) or serum insulin (Fig. 3H) across the experimental groups.

**Fig. 4:**
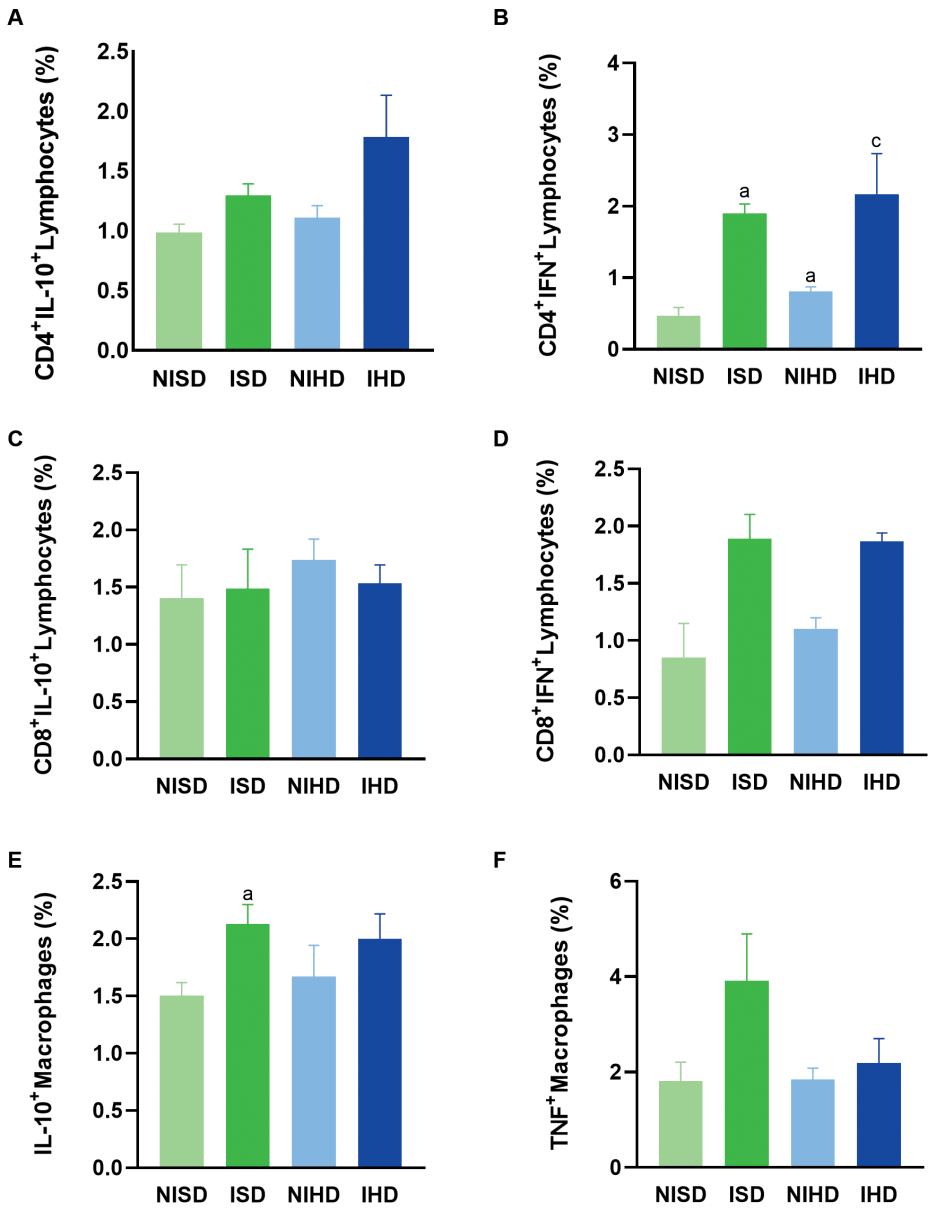
immunophenotyping of splenic mononuclear cells in the following groups: non-infected animals subjected to a standard diet (NISD), *Trypanosoma cruzi*-infected animals subjected to a standard diet (ISD), non-infected animals subjected to a hyperglycaemic diet (NIHD), and *T. cruzi*-infected animals subjected to a hyperglycaemic diet (IHD). (A) Percentage of CD4⁺ IL-10⁺ T lymphocytes; no statistically significant differences were observed between the groups. (B) Percentage of CD4⁺ IFN-γ⁺ T lymphocytes; the letters “a” and “c” indicate statistically significant differences compared with the control group (NISD) and the non-infected group subjected to the hyperglycaemic diet (NIHD), respectively. (C) Percentage of CD8⁺ IL-10⁺ T lymphocytes; no statistically significant differences were observed between the groups. (D) Percentage of CD8⁺ IFN-γ⁺ T lymphocytes; no statistically significant differences were observed between the groups. (E) Percentage of IL-10⁺ macrophages; the letter “a” denotes a statistically significant difference compared with the control group (NISD). (F) Percentage of TNF⁺ macrophages; no statistically significant differences were observed between the groups. Values are expressed as mean ± standard deviation.

*Immunophenotyping of splenic mononuclear cells* - On day 10 post-infection, there were no significant differences in the percentage of IL-10-producing CD4⁺ (Fig. 4A) or CD8⁺ T lymphocytes (Fig. 4C), nor in the percentage of IFN-γ-producing CD8⁺ T lymphocytes (Fig. 4D). The percentage of IFN-γ-producing CD4⁺ T lymphocytes was higher in both infected groups (ISD and IHD) when compared with their respective non-infected control groups (NISD and NIHD) (Fig. 4B).

Regarding IL-10-producing macrophages, the percentage was higher in the ISD group compared with the NISD group (Fig. 4E). However, no significant differences were observed in the percentage of TNF-producing macrophages between the groups (Fig. 4F).

**Fig. 5:**
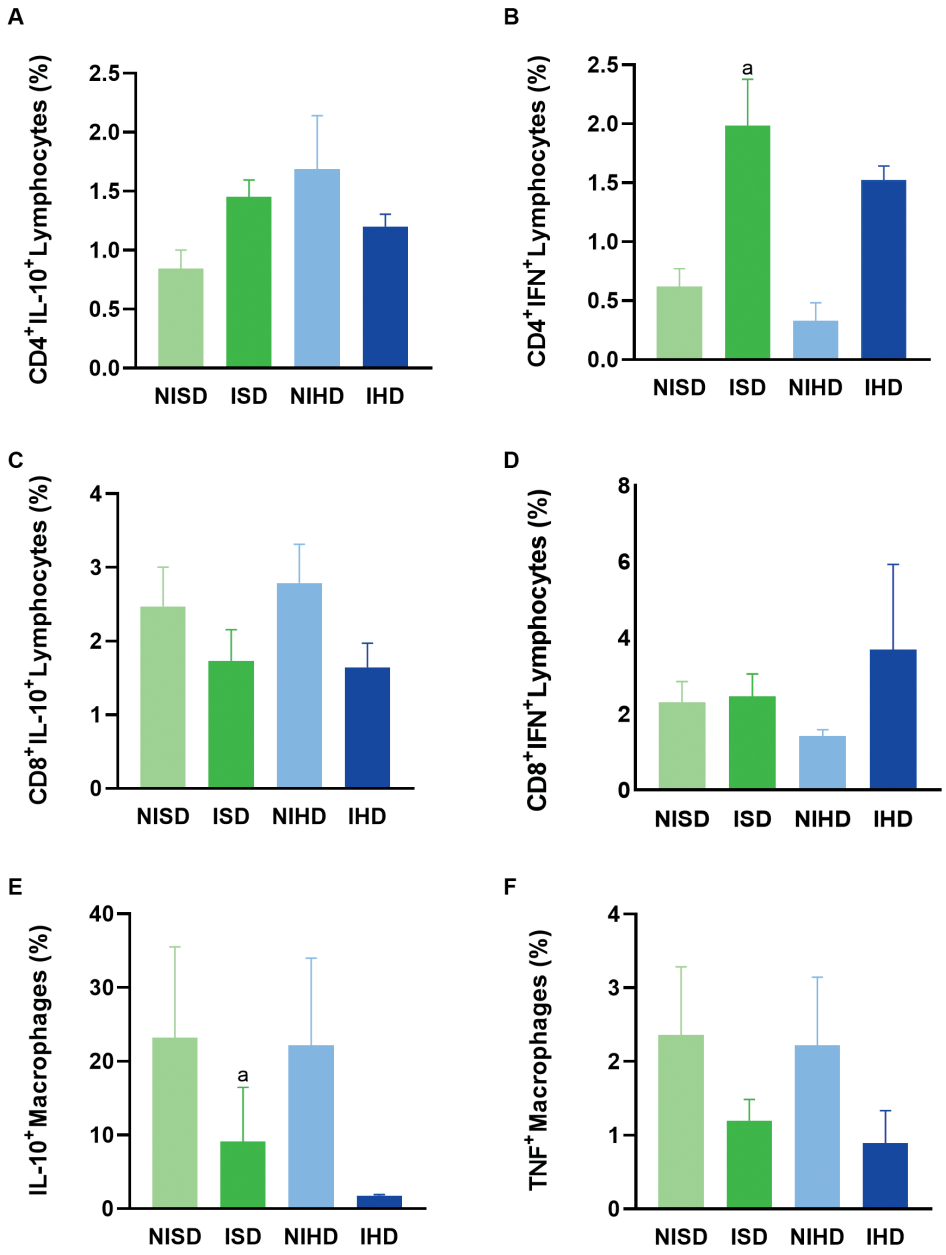
phenotyping of splenic mononuclear cell profiles in C57BL/6 mice in the following groups: non-infected animals subjected to a standard diet (NISD), *Trypanosoma cruzi*-infected animals subjected to a standard diet (ISD), non-infected animals subjected to a hyperglycaemic diet (NIHD), and *T. cruzi*-infected animals subjected to a hyperglycaemic diet (IHD). (A) Percentage of CD4+ IL-10+ T lymphocytes; no statistically significant differences were observed between the groups. (B) Percentage of CD4+ IFN-γ+ T lymphocytes; the letter “a” indicates a significant difference from the control group (NISD). (C) Percentage of CD8+ IL-10+ T lymphocytes; no statistically significant differences were observed between the groups. (D) Percentage of CD8+ IFN-γ+ T lymphocytes; no statistically significant differences were observed between the groups. (E) Percentage of IL-10+ macrophages; no statistically significant differences were observed between the groups. (F) Percentage of TNF+ macrophages; no statistically significant differences were observed between the groups. Values are expressed as mean ± standard deviation.

On day 30 post-infection, the percentages of IL-10-producing CD4⁺ and CD8⁺ T lymphocytes (Fig. 5A, C), as well as IFN-γ-producing CD8⁺ T lymphocytes (Fig. 5D), did not show statistically significant differences between the groups. However, the percentage of IFN-γ-producing CD4⁺ T lymphocytes was higher in the ISD group compared with the NISD group (Fig. 5B).

Additionally, the percentage of IL-10-producing macrophages was smaller in the ISD group compared with its control (NISD) (Fig. 5E). In contrast, no significant differences were observed in the percentages of CD11⁺ macrophages producing TNF among the groups (Fig. 5F).

## DISCUSSION

Since CD remains a globally neglected condition with significant public health impacts, and metabolic disorders are highly prevalent in Brazil, conducting a study that explores the effects of a hyperglycaemic diet on *T. cruzi* infection in an experimental model may be useful for understanding the potential interactions between metabolic alterations and parasitic infections.

In our study, mice fed a hyperglycaemic diet exhibited higher glycaemic levels at the early time point (0 DAI), in agreement with a previous study.^18^ However, at 30 DPI (DAI), infected animals under a hyperglycaemic diet showed reduced blood glucose levels, likely related to infection-associated symptoms such as lethargy and reduced food intake. Combs et al.^19^ previously observed that mice infected with *T. cruzi* and subjected to a high-glucose diet showed hypoglycaemia, suggesting this may result from cytokine storms that suppress appetite and/or increase glucose uptake by the parasite.^11^ ^,^ ^20^ Although amastigotes preferentially utilise amino acids and fatty acids as carbon sources, *T. cruzi* can also modulate host cell metabolism to facilitate glucose uptake, as demonstrated by Shah-Simpson.^21^

Hyperglycaemic diet intake also led to increased body weight and adipose tissue mass, particularly in non-infected groups. These findings are consistent with previous studies using carbohydrate-rich diets in rodents.^22^ ^,^ ^23^ In infected groups, adipose tissue mass was reduced at 30 DAI, probably due to lower food intake. Despite known associations between hyperglycaemia and increased cholesterol levels, infected animals under the hyperglycaemic diet showed reduced total cholesterol, potentially reflecting the impact of parasitaemia on appetite and lipid metabolism. Inflammation and infection are known to induce marked changes in lipid profiles, particularly reductions in total cholesterol, LDL-C, and HDL-C, as part of the host defence response.^24^ ^,^ ^25^

Regarding the immune response, higher percentages of IFN-γ-producing CD4⁺ T lymphocytes were observed at 10 and 30 DAI, consistent with the inflammatory profile expected during acute *T. cruzi* infection.^26^ ^,^ ^27^ Interestingly, non-infected mice under a hyperglycaemic diet also showed increased IFN-γ expression, possibly due to diet-induced inflammation. This aligns with reports that obesity enhances IFN-γ production and the antigen presentation capacity of adipocytes.^28^ ^,^ ^29^

Macrophages are central to early immune responses against *T. cruzi*, particularly via M2 polarisation which enhances phagocytosis and parasite clearance.^30^ ^,^ ^31^ In our study, infected animals under a standard diet showed increased macrophage populations, suggesting a host attempt to control parasitaemia.

Cardiac parasitic load was higher at 30 DAI compared with 10 DAI, particularly in animals infected with the Colombian strain of *T. cruzi*, known for its cardiac tropism.^32^ Chronic parasitism in the heart promotes persistent inflammation, necrosis, and fibrosis, ultimately leading to progressive myocardial damage, as previously demonstrated in both human and murine studies.^33^ ^,^ ^34^ ^,^ ^35^

Adipose tissue also emerged as a relevant site of parasitic persistence. In our study, mice on a hyperglycaemic diet showed higher adipose tissue parasitic loads at day 10 compared with day 30 post-infection. It was demonstrated in a murine model that *T. cruzi* infects adipose tissue early and remains detectable into the chronic phase. Moreover, DNA from *T. cruzi* was identified by polymerase chain reaction (PCR) in adipose tissue from three out of ten seropositive patients with cardiomyopathy and conduction defects.^36^ Likewise, found viable parasites in adipose tissue even after 300 days of infection, with parasite loads comparable to those in the heart. This persistence may be explained by the slow turnover of adipocytes, which are long-lived and metabolically active cells.^19^

The ability of *T. cruzi* to persist in adipose tissue has important implications for treatment. The parasite’s presence in a low-turnover, poorly vascularised tissue may reduce the efficacy of benznidazole and facilitate immune evasion. Such characteristics are not exclusive to CD; adipose tissue has been shown to serve as a reservoir in other infections, including Brill-Zinsser disease,^37^ tuberculosis,^38^ and malaria.^39^

In the context of HIV, both the infection and antiretroviral therapy may induce lipodystrophy, characterised by reduced subcutaneous fat and redistribution of fat to central depots. This remodelling of adipose tissue could lead to the release of intracellular *T. cruzi* into circulation, favouring disease reactivation in immunocompromised patients.^40^

Together, our findings reinforce the role of adipose tissue as an active participant in the immunometabolic alterations observed during *T. cruzi* infection. Its function as a parasitic reservoir and immune modulator underscores the need to consider metabolic status and tissue-specific parasite dynamics when evaluating treatment efficacy and disease progression.

The results of this study, highlighting adipose tissue as a potential reservoir for *T. cruzi*, the high cardiac parasite load during the late acute phase, and the occurrence of hypoglycaemia in infected animals, underscore the complex interaction between metabolic and infectious processes. Extrapolating from the murine model to human disease, these findings emphasise the need for more comprehensive clinical monitoring of Chagas patients, particularly those with metabolic comorbidities. Regular assessment of lipid profiles, glycaemic status, and muscle mass may be essential for improved management and prognosis in chronic CD, especially in the context of altered metabolism and potential therapeutic failure.

*In conclusion* - The results indicate that infection with *T. cruzi* was either directly or indirectly responsible for the observed metabolic alterations, and that consumption of a hyperglycaemic diet significantly influenced parasitological parameters during the acute phase of infection.

## Data Availability

The contents underlying the research text are included in the manuscript.
